# An Integrated Pipeline and Overexpression of a Novel Efflux Transporter, YoeA, Significantly Increases Plipastatin Production in *Bacillus subtilis*

**DOI:** 10.3390/foods13111785

**Published:** 2024-06-06

**Authors:** Mengxi Wang, Jie Zheng, Sen Sun, Zichao Wu, Yuting Shao, Jiahui Xiang, Chenyue Yin, Rita Cindy Aye Ayire Sedjoah, Zhihong Xin

**Affiliations:** Key Laboratory of Food Processing and Quality Control, College of Food Science and Technology, Nanjing Agricultural University, Nanjing 210095, China; 2020208023@stu.njau.edu.cn (M.W.); 2021108049@stu.njau.edu.cn (J.Z.); 2021108048@stu.njau.edu.cn (S.S.); 2021121005@stu.njau.edu.cn (Z.W.); 2018208019@njau.edu.cn (Y.S.); 2021208022@stu.njau.edu.cn (J.X.); 2022108016@stu.njau.edu.cn (C.Y.); ritacindy19@gmail.com (R.C.A.A.S.)

**Keywords:** *Bacillus subtilis*, plipastatin, antimicrobial lipopeptide, efflux transporter (YoeA), lipopeptide production

## Abstract

Plipastatin, an antimicrobial peptide produced by *Bacillus subtilis*, exhibits remarkable antimicrobial activity against a diverse range of pathogenic bacteria and fungi. However, the practical application of plipastatin has been significantly hampered by its low yield in wild *Bacillus* species. Here, the native promoters of both the plipastatin operon and the *sfp* gene in the mono-producing strain M-24 were replaced by the constitutive promoter P_43_, resulting in plipastatin titers being increased by 27% (607 mg/mL) and 50% (717 mg/mL), respectively. Overexpression of long chain fatty acid coenzyme A ligase (LCFA) increased the yield of plipastatin by 105% (980 mg/mL). A new efflux transporter, YoeA, was identified as a MATE (multidrug and toxic compound extrusion) family member, overexpression of *yoeA* enhanced plipastatin production to 1233 mg/mL, an increase of 157%, and knockout of *yoeA* decreased plipastatin production by 70%; in contrast, overexpression or knockout of *yoeA* in mono-producing surfactin and iturin engineered strains only slightly affected their production, demonstrating that YoeA acts as the major exporter for plipastatin. Co-overexpression of *lcfA* and *yoeA* improved plipastatin production to 1890 mg/mL, which was further elevated to 2060 mg/mL after *abrB* gene deletion. Lastly, the use of optimized culture medium achieved 2514 mg/mL plipastatin production, which was 5.26-fold higher than that of the initial strain. These results suggest that multiple strain engineering is an effective strategy for increasing lipopeptide production, and identification of the novel transport efflux protein YoeA provides new insights into the regulation and industrial application of plipastatin.

## 1. Introduction

Antimicrobial peptides (AMPs) constitute a class of small-molecule peptides that are widely present in nature, and which typically comprise fewer than 100 amino acids. They aid the body in defending against external microorganisms and constitute an integral component of the innate immune system [1]. It is generally believed that AMPs primarily disrupt the integrity of microorganism cell membranes or cell walls to inhibit or kill microorganisms. Recent studies have also revealed that the mechanism of action of AMPs may additionally target intracellular sites directly to eliminate cells through non-membrane-damage mechanisms [2].

Plipastatin, also known as fengycin [3], is a cyclic lipopeptide synthesized by several species of *Bacillus*. It consists of a ten-amino acid-core frame linked with a C14–C21 β-hydroxy fatty acid (β-OH-FA) side chain [4]. Plipastatin shows diverse biological activities, with antibacterial [5], antiviral [6], and antitumor [7] properties highlighting its substantial potential for various applications, including biological control [8], post-harvest preservation of fruits and vegetables [9], and biomedicine. Like daptomycin, which has been commercialized and used in clinics, the interaction between plipastatin and phospholipid bilayers is also highly dependent on both concentration and lipid composition. The main difference is that plipastatin has a strong interaction with the fungal cell membrane, whereas daptomycin exerts a potent effect on the Gram-positive bacterial membrane [10]. Furthermore, plipastatin can eradicate colonized methicillin-resistant *Staphylococcus aureus* (MRSA) in the gastrointestinal tract by effectively blocking the quorum-sensing system [11], suggesting a probiotic-based method for *S*. *aureus* decolonization and new approaches to fighting *S*. *aureus* infection.

In theory, plipastatin can be synthesized either through chemical means or by fermentation of specific *Bacillus* spp. However, the chemical synthesis of plipastatin remains challenging owing to its intricate and complex molecular structure, whereas the production of plipastatin by wild *Bacillus* spp. commonly results in a very low yield, in most cases less than 100 mg/L [12,13]. Furthermore, microbial fermentation and the following purification processes are labor-intensive and time-consuming, substantially limiting the practical application of plipastatin. Hence, it is of great importance to develop new or integrated strategies to enhance plipastatin production. Consequently, a large number of studies have been performed to enhance the production of plipastatin through various pipelines, including promoter engineering [14], knockout of competing pathways [15], increasing supplementation with precursor substrates [12], optimizing fermentation parameters [16], and overexpressing specific exporter proteins [17].

Replacement of the native plipastatin promoter with the constitutive P*_veg_* promoter increased the production of plipastatin to 174.63 mg/L in the engineered strain BSf04 [18]. Similarly, the replacement of the P*_pps_* promoter in *Bacillus. subtilis* 168 with the *Bacillus. amyloliquefaciens* P*_amyQ_* promoter resulted in plipastatin production of 452 mg/L [19]. Amino acids and fatty acids are essential precursors in the synthesis of plipastatin. The *birA*, *acs*, and *accACD* genes are associated with fatty acid synthesis, and overexpression of these genes in the 168DS strain increased plipastatin production 1.37-fold [12]. Similarly, overexpression of biotin carboxylase II (YngH) resulted in a 145% enhancement in surfactin production by preserving the activity of acetyl-coenzyme A carboxylase (ACCase) [20].

Loading of fatty acid side chains is a crucial step in cyclic lipopeptide synthesis. Fatty acid acyl-CoA ligase is a key enzyme that activates 3-hydroxy fatty acids to facilitate the synthesis of cyclic lipopeptides. *B. subtilis* fatty acyl-CoA ligase (LcfA) plays a pivotal role in the activation of 3-hydroxy fatty acids and contributes to the biosynthesis of surfactin. Deletion of the *lcfA* gene results in a significant 45.6% reduction in surfactin production, underscoring the positive impact of LcfA’s enzymatic activity on enhancing surfactin yield. Given the analogous structure and synthesis pathways, as well as the shared precursors of plipastatin and surfactin, it is speculated that the plipastatin fatty acid loading mechanism is similar to that of surfactin, and thus enhancing *lcfA* gene expression might increase plipastatin production [21].

During microbial evolution, microorganisms have developed mechanisms to mitigate the potentially harmful effects of their self-produced secondary metabolites. Efflux pumps represent a major means to relieve this self-inhibition through transporting secondary metabolites extracellularly [22]. Overexpression of the proton motive force (PMF)-dependent transporter YerP results in a remarkable 145% increase in surfactin production [23]. However, the efflux pathways remain unknown for other lipopeptides in *B. subtilis*, and, thus far, no genes encoding a plipastatin efflux transporter have been identified. Additionally, the fermentation parameters—such as pH, temperature, agitation speed, oxygen supply, and medium composition—are all important factors that have significant effects on the yield of lipopeptide production [16].

Herein, to address the challenge of insufficient plipastatin production in practice, we have used an integrated approach—including replacing the natural promoters with strong promoters, overexpression of the *lcfA* gene, bioinformatics analysis, identification of a pathway-specific effluent transporter of plipastatin, knocking out the global negative regulator AbrB, and optimizing the medium components—to increase plipastatin production.

## 2. Materials and Methods

### 2.1. Strains, Plasmids, and Culture Conditions

The strains used in this study are shown in Table S1. *B. subtilis* M-24 (M-24) is a mono-producing plipastatin engineering strain constructed by our laboratory. *Escherichia coli* DH5α was used for plasmid construction and propagation. *B. subtilis* and *E. coli* were cultured overnight in Luria-Bertani (LB) medium, containing 1% tryptone, 0.5% yeast extract, and 1% NaCl on a 37 °C, 180 rpm shaker. Transformants in *E. coli* were selected on LB plates containing ampicillin (100 μg/mL) and kanamycin (30 μg/mL for pJOE8999 and its derivatives, 50 μg/mL for pJMP-Cm derivatives), whereas *B. subtilis* was grown in LB broth supplemented with spectinomycin (100 μg/mL), kanamycin (7.5 μg/mL), and chloramphenicol (5 μg/mL).

Restriction endonucleases *Sfi*I (R0123V) and *Bsa*I (R3733V) were purchased from New England Biolabs (NEB, Ipswich, MA, USA). The DL2000 DNA Marker was purchased from TaKaRa (Beijing, China). The FastPure Plasmid Mini Kit (DC201-01), the FastPure Gel DNA Extraction Mini Kit (DC301-01), T4 DNA ligase, the ClonExpress II One Step Cloning Kit, and 2 × Rapid Taq Master Mix (P222-01) were purchased from Vazyme (Nanjing, Jiangsu, China).

### 2.2. Plasmid and Strain Construction

All plasmids and primers used in this study are listed in Tables S2 and S3. To determine promoter activity, endogenous promoters from *B. subtilis*, including P_43_, P*_veg_*, P*_sigA_*, P*_amy_*, P*_xyl_*, P*_manP_*, P*_gsi_*, P*_ohrB_*, P*_aprE_*, and P*_rpsF_*, were fused with the *lacZ* reporter gene to generate 10 recombinant plasmids (e.g., pJM-P_43_-*lacZ*), and the lengths of the different promoters were determined by Biocyc (https://bsubcyc.org/) accessed on 15 October 2022. Then, these plasmids were transformed into the M-24 strain through the natural-transformation-competence method, resulting in 10 different recombination strains with the expression of β-galactosidase activity under different promoters [16], with the M-24 strain as a control.

### 2.3. Construction of Plipastatin-Promoter-Replacement Strains Using CRISPR/Cas9 Technology

The *E. coli*–*B. subtilis* shuttle plasmid pJOE8999 was used as the initiation plasmid to replace the native promoter of plipastatin and the *sfp* operon with P_43_. A double-stranded sgRNA (*sfp*-sgRNA or *ppsA*-sgRNA) fragment was obtained by annealing two reverse-complementary single-stranded nucleic acids. This fragment was cloned into pJOE8999 plasmid via *Bsa* I (type II restriction endonuclease)-mediated Golden Gate assembly. The upstream homologous arm, the downstream homologous arm fused with the P_43_ promoter at the N-terminal end, and the plasmid with sgRNA were digested by *Sfi* I restriction endonuclease and ligated with T4 DNA ligase to obtain the pJOE8999-P_43_-*pps*A and pJOE8999-P_43_-*sfp* recombinant plasmids for promoter replacement. The plasmids were verified by PCR using primers Ts-F/R and Tp-F*/*R and transferred into *B. subtilis* competent cells. The transformed cells were coated onto LB solid medium containing spectinomycin, kanamycin, and 0.3% D-mannose, and single colonies grew on the solid plates for 18–36 h. The positive recombination strains were checked by colony PCR using the primers Vs-F/R and Vp-F*/*R, and the resulting cells were streaked onto LB plates without antibiotics and incubated at 50 °C to cure the plasmids.

### 2.4. Construction of Gene Overexpression and Knockout Plasmids

The *E. coli–B. subtilis* shuttle plasmid pJMP-Cm was used to construct the integrated plasmid for gene overexpression. Primers used to amplify the *lcfA*, *yngH*, *srf*P, and *yoe*A genes from the M-24 genome are shown in Table S3. For the *lcfA*, *yoeA,* and *yngH* genes, PCR amplification products were purified by agarose electrophoresis, digested by *Spe* I and *EcoR* I, and then ligated to pJMP-Cm, which was digested by the same restriction endonuclease. The *srf*P gene was inserted into pJMP-Cm at the *Spe* I and *EcoR* I restriction sites by infusion cloning. To create the pJM-*yoeA*-*lcfA* plasmid, the *yoeA* and *lcfA* gene fragments were assembled by overlapping PCR and then inserted between the *Spe* I and *EcoR* I restriction sites in pJMP-Cm. The ligation mixture was transformed into *E. coli* DH5a competent cells to obtain the recombination plasmids pJM-*lcfA*, pJM-*yoeA*, and pJM-*yng*H.

For the gene knockout plasmid construction, the upstream and downstream regions flanking the target gene were amplified by PCR. The chloramphenicol and kanamycin resistance genes were amplified from pJMP-Cm and pJOE8999, respectively. The plasmid backbone was amplified by the pJMP-Cm vector. These fragments were ligated with an infusion clone to obtain the gene knockout plasmid. The colonies of *E. coli* that were positive for ampicillin resistance were verified by PCR and then transformed into *B. subtilis*. The resulting positive clones were coated onto LB solid medium containing the corresponding resistance and verified by colony PCR.

### 2.5. Cultivation Engineering Strains and Extraction Plipastatin

Recombinant strains were inoculated into seed culture media and shaken at 37 °C for 20 h. Subsequently, 2.5 mL of the pre-culture was transferred to 50 mL of fermentation medium (1 L modified Landy medium containing 2 g L-glutamine, 40 g glucose, 2.3 g sulfuric acid, 1 g potassium hydrogen phosphate, 0.5 g potassium chloride, 0.5 g magnesium sulfate, 1.6 mg sulfuric acid,1.2 mg iron sulfate, 0.4 mg sulfuric acid, and 20.927 g MOPS; pH 7.0–7.2), and shaken at 33 °C for 7 days.

The culture broth was centrifuged at 4 °C and 10,000× *g* for 20 min then the supernatant was collected. The pH of the supernatant was adjusted to 2 using 6 M hydrochloric acid, followed by incubation at 4 °C overnight for the complete precipitation of plipastatin. The next day, the precipitate was centrifuged and re-suspended in methanol, the pH of the solution was adjusted to 7, and complete dissolution was assisted by ultrasonic cleaners. The supernatant was then evaporated under a vacuum with a rotary evaporator at 40 °C, and the final product was dissolved in 1 mL of methanol.

### 2.6. Quantitative Analysis of Plipastatin

The crude extract was separated into two fractions (Fr1 and Fr2) by a Sephadex LH-20 gel column using CHCl_3_:MeOH (1:1, *v*/*v*) as the eluent; Fr1 was further purified by preparative liquid chromatography with an Agilent ZORBAX SB-C18 column (9.4 mm × 250 mm, 5 μm, Agilent, Santa Clara, CA, USA). Fr1 was purified by reversed-phase HPLC (Shimadzu LC-20AT, ZORBAX SB-C18, 9.4 mm × 250 mm, 5 μm, Agilent) using a gradient solvent system from 50% to 90% CH_3_CN:H_2_O (1‰ trifluoroacetic acid) over 25 min to yield pure plipastatin 1 (4.0 mg, tR = 20.5 min). Fr2 was purified with the same HPLC method to yield plipastatin 2 (1 mg, tR = 19.2 min). Plipastatins in samples were analyzed by an external standard method using plipastatin 1 as a standard sample. For HPLC analysis (Waters 2998, Milford, MA, USA USA), 20 μL of crude extract was filtered through a 0.22 μm organic filter and injected into the Agilent RTC18 column (4.6 × 150 mm, 5 μm, Agilent, USA) with a flow rate of 0.8 mL/min. Mobile phase A consisted of water and 0.1% trifluoroacetic acid, whereas mobile phase B was acetonitrile supplemented with 0.1% trifluoroacetic acid. The gradient elution used was 50−95% B over 35 min, 95% B over 10 min, 95−50% B over 10 min, and 50% B over 5 min. Peaks eluting from the column were monitored by UV detection at 214 nm, and the column temperature was maintained at 30 °C.

### 2.7. Determining the Antibacterial Activity of Plipastatin

The antimicrobial activities of the plipastatin extracts were evaluated using the diffusion gel method as described by Aktuganov et al. [24]. Four pathogenic bacteria, including *E. coli* (CICC10389), *Clostridium perfringens* (CICC22949), *Staphylococcus aureus* (ATCC25923), *Micrococcus tetragenus* (ATCC 35098), and one fungal pathogen *Clostridium perfringens* (ATCC 90028) were used as test microorganisms. The extraction from the fermentation broth of each recombination strain was performed according to the method described above in Section 2.5, and each pore (diameter 9 mm) was filled with 10 μL of extraction solution. The plipastatin extract was dissolved in methanol to form a stock solution. Methanol was also used as a control solvent. Antibacterial activity was tested on an LB plate at 37 °C for 5–12 h, and antifungal activity was tested on potato dextrose agar medium with culturing at 30 °C for 72 h. The zones of inhibition (mm in diameter) were tested and recorded.

## 3. Results

### 3.1. Impact of Promoter Replacement on Plipastatin Production

**Figure 1:**
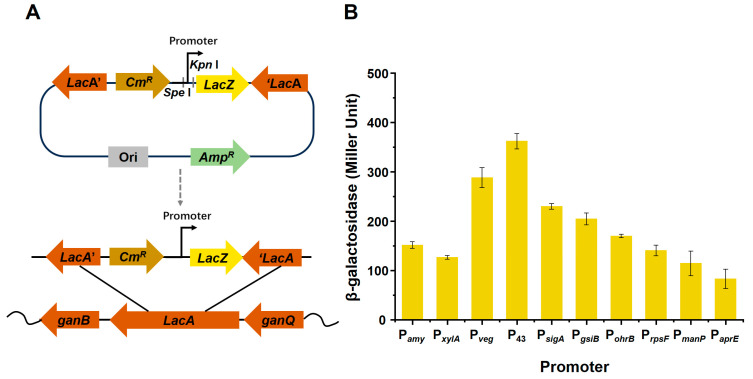
Construction of promoter-recombinant strains and analysis of different promoter activities. (**A**) Schematic diagram of the fusion of different promoters and the *lacZ* gene in recombinant bacteria. (**B**) β-galactosidase activities of different promoters.

Promoter replacement is an effective strategy to enhance target-product yield. Ten promoters from the *B. subtilis* genome were amplified by PCR and fused with the β-galactosidase gene in the pJMP-cm plasmid. The ten promoters included the promoter for the amylase gene, P*_amy_*, the promoter for the xylose operon, P*_xylA_*, the promoter for the veg gene, P*_veg_*, the promoter for the cytidine deaminase gene, P_43_, the promoter for the general stress protein glucose starvation-induced gene, P*_gsiB_*, the promoter for the organic hydroperoxide resistance reductase B gene, P*_ohrb_*, the promoter for the ribosomal protein S6 gene, P*_rpsF_*, the promoter for the phosphotransferase system (PTS) mannose-specific enzyme IIBCA component gene, P*_manP_* and the promoter for the serine alkaline protease gene, P*_aprE_*. The constructs of recombinant strains fused with different promoters are shown in Figure 1A. The promoter strength was quantified by β-galactosidase activity when the strains were cultured to the logarithmic growth phase in a modified Landy medium, as shown in Figure 1B. All promoters exhibited different levels of activity, among which the P_43_ promoter showed the highest activity with a value of 331.96 U (Figure 1B), whereas the P*_aprE_* promoter had the lowest activity (82.69 U). P_43_, which transcribes the cytidine deaminase gene (*cdd*) and acts as a strong constitutive promoter in *B. subtilis*, can be recognized by σ^A^ and σ^B^ and is responsible for the transcription of housekeeping and stress-related genes [25]. Thus, we selected this promotor to replace the natural plipastatin promoter.

**Figure 2:**
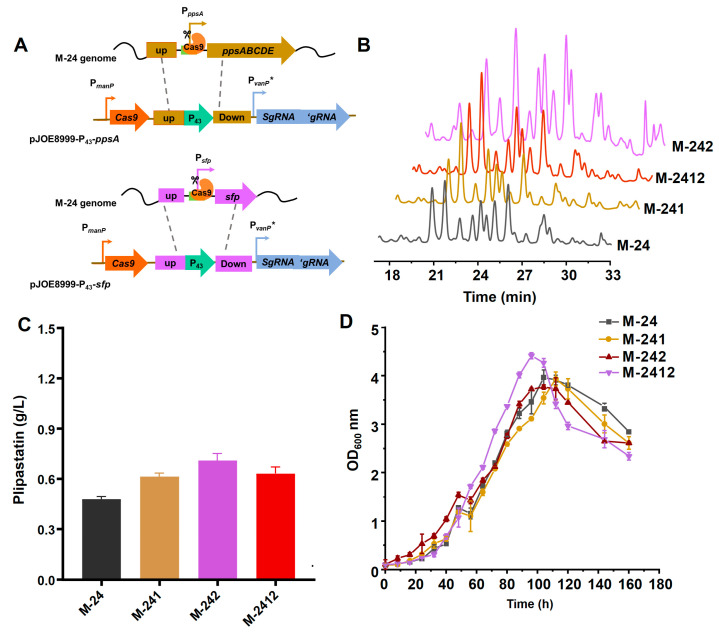
Effect of promoter replacement on plipastatin production. (**A**) Schematic diagram of the M-24 natural promoter replacement strategy, based on CRISPR/Cas9 technology. (**B**) HPLC profiles of the promoter-replacement engineered strains. (**C**) Comparison of plipastatin production in the promoter-replacement engineered strains. (**D**) Growth curves of promoter-replacement engineered strains.

The native promoters of the plipastatin operon and the *sfp* gene were individually replaced by the P_43_ promoter using the CRISPR/Cas9 system, as shown in Figure 2A. *sfp* encodes a 4′-phosphopantetheinyl transferase (26 kDa) that catalyzes the transfer of the 4′-phosphopantetheine from coenzyme A to the conserved serine residue of the peptidyl carrier protein (PCP), which has been demonstrated to be an essential participant in the biosynthesis of non-ribosomal peptides. The resulting recombinant strains, M-241 and M-242, were obtained and fermented for 7 days, and then plipastatin production was analyzed by high-performance liquid chromatography (HPLC), as shown in Figure 2B. The HPLC profiles revealed that the retention times of the plipastatin peaks from the two recombinant strains were between 17 and 33 min, similar to those for the M-24 strain, but with significantly increased peak areas (Figure 2B). The yields of M-241 and M-242 were increased 1.27-fold (607 mg/mL) and 1.5-fold (717 mg/mL), respectively. Simultaneous replacement of the promoters of plipastatin and the *sfp* gene with the P_43_ promoter, resulting in strain M-2412, gave a plipastatin yield of 625 mg/mL, which was slightly higher than that for M-241 but lower than that for M-242, as shown in Figure 2C.

To determine whether the growth of these three recombinant strains was affected, the strains were fermented and the growth curves were measured and plotted, as shown in Figure 2D. Strains M-24, M-241, and M-242 all reached a maximum optical density of 600 nm (OD_600_) at 104 h. For the M-2412 strain, the maximum OD_600_ occurred at 96 h, eight hours sooner than M-24, and the OD_600_ sharply decreased at 104 h, indicating partial cell death during fermentation. This might be attributed to rapid cell proliferation causing a swift depletion of nutrients in the culture medium, which in turn would disrupt the balance of the cellular metabolic network, resulting in a reduction in plipastatin production.

### 3.2. Impact of Fatty Acid Supply on Plipastatin Production

**Figure 3:**
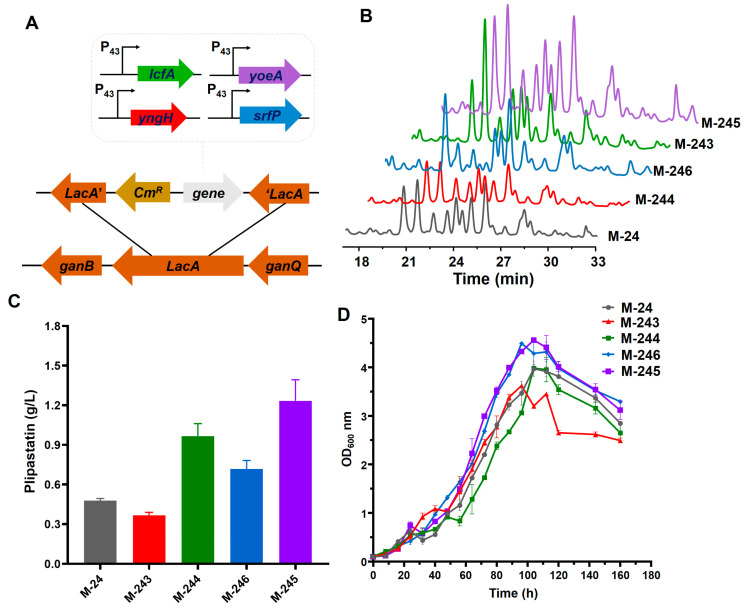
Effect of gene overexpression on plipastatin production. (**A**) Schematic diagram of the construction of recombinant strains. (**B**) HPLC fingerprint of the recombinant strains. (**C**) Comparison of plipastatin production of recombinant strains. (**D**) Growth curves of the recombinant strains.

As essential precursors, fatty acids are particularly important for the synthesis of plipastatin [18]. For instance, plipastatin production increased by 2.12 times, 3.03 times, 2.60 times, and 1.77 times in *B. amyloliquefaciens* Pc3 when myristic acid, pentadecanoic acid, heptadecanoic acid, and nonadecanoic acid, respectively, were individually added to the culture medium [26]. Fatty acid biosynthesis is initiated by acetyl-CoA carboxylase in the cytoplasm, which is a rate-limiting enzyme that converts acetyl-CoA into malonyl-CoA [27]. The *yngH* gene encodes a subunit of ACCase and plays a crucial role, compared with other subunits, in maintaining ACCase [20]. Overexpression of YngH might increase the fatty acid supplement and enhance the plipastatin titer. The construction of the *yngh* gene overexpression strain is shown in Figure 3A and plipastatin production was analyzed by high-performance liquid chromatography (HPLC) (Figure 3B). However, recombinant strain M-244, constructed by overexpressing the *yngH* gene in M-24, exhibited a 23% reduction in plipastatin production (Figure 3C). The result is opposite to a report wherein overexpression of the *yngH* gene led to increased surfactin production, but it is consistent with a report in which the *ldeHA* gene (synonymous with *yngH*) was overexpressed in *B. subtilis* BSF04-2 [18]. According to the growth curve (Figure 3D), the maximum OD_600_ of strain M-244 was lower than that of M-24, and the time required to reach the maximum OD_600_ was reduced by eight hours, suggesting that overexpression of *yngH* might disrupt the balance between biotin carboxylase and carboxyl transferase, thereby inhibiting the growth of the strain and subsequently reducing plipastatin production.

Acyl-CoA ligases in *B. subtilis* activate 3-hydroxy fatty acids to form 3-hydroxy fatty acyl-CoA, which is recognized as a substrate for the initiation of surfactin synthesis [28,29]. Biochemical studies on four putative acyl-CoA ligases in *B. subtilis* revealed that LcfA can activate 3-hydroxy fatty acids for surfactin biosynthesis in vitro, and that disruption of the *lcfA* gene is detrimental to surfactin production [23]. The fatty acid-loading mechanism of plipastatin is believed to be similar to that of surfactin, and we hypothesized that overexpression of the *lcfA* gene could increase the plipastatin titer. The construction of the gene-overexpression recombinant strain is shown in Figure 3A, and plipastatin production was analyzed by high-performance liquid chromatography (HPLC) (Figure 3B). The *lcfA* gene was cloned and integrated into the *lacA* site, resulting in recombinant strain M-243, for which plipastatin production was increased from 478 to 980 mg/L, which was 2.05-fold higher than that for M-24 (Figure 3C). The growth curve of strain M-243 was similar to that of M-24 (Figure 3D), suggesting that the overexpression of *lcfA* did not affect strain growth.

### 3.3. Impact of Overexpressed Transport Proteins on Plipastatin Production

In *B. subtilis*, efflux transporters are membrane-integrated proteins that export endogenous secondary metabolites, such as lipopeptides, antibiotics, and toxic metals, to prevent self-intoxication, reduce feedback inhibition, and increase metabolite production [30]. YerP, a member of the major facilitator superfamily (MFS) transporter family in *B. subtilis*, functions to transport multiple substrates, including amino acids, lipids, and both inorganic and organic ions [31]. YerP plays a major role in surfactin efflux [32]. The plipastatin yield in the recombinant strain M-246, generated by overexpression of the *srfP* (Figure 3A,B) (homologous gene *yerP*) gene in the M-24 strain, increased to 670 mg/mL, a 1.65-fold improvement (Figure 3C), demonstrating that SrfP can also transport and efflux plipastatin.

**Figure 4:**
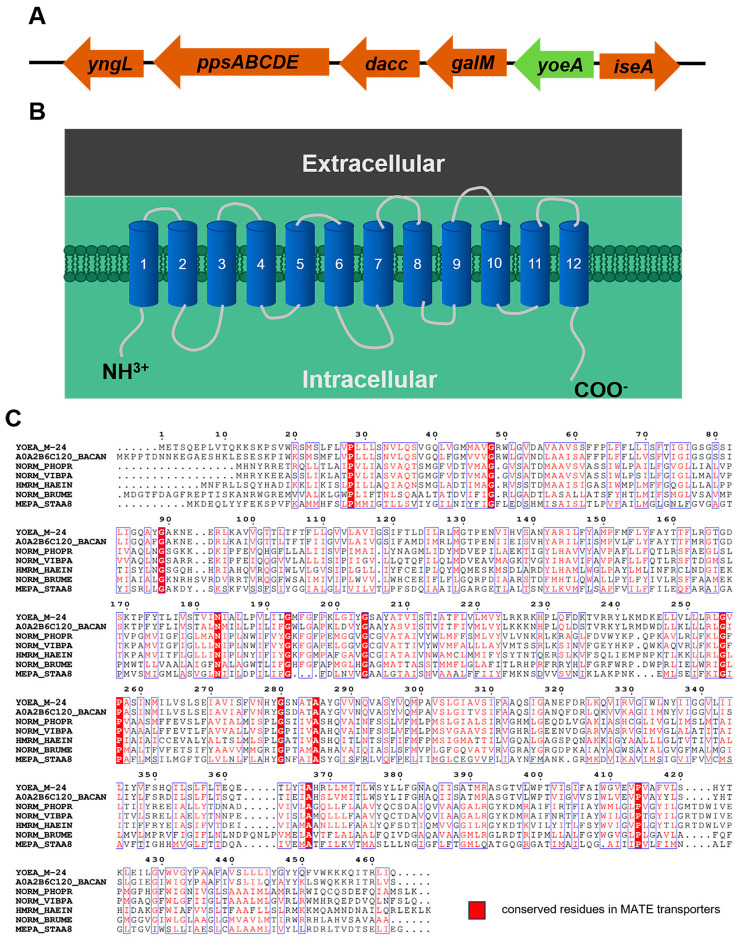
Bioinformatics analysis of YoeA protein. (**A**) Location of the *yoeA* gene and the plipastatin synthesis gene cluster. (**B**) Predicted topology diagram of YoeA transporters in *B. subtilis* M-24. (**C**) Sequence alignment of the MATE transporters YoeA_M-24 (UniProt ID: O34474), A0A2B6C120_BACAN (UniProt ID: A0A2B6C120), NORM_PHOPR (UniProt ID: Q6LQ49), NORM_VIBPA (UniProt ID: O82855), HMRM_HAEIN (UniProt ID: P45272), NORM_BRUME (UniProt ID: Q8YFD7), and MEPA_STAA8 (UniProt ID: Q2G140).

Analysis of the flanking sequence of the plipastatin operon (Table 1) revealed a protein with an unknown function, YoeA, located 2767 bp upstream of the *ppsA* operon (Figure 4), which might encode a putative Na^+^-driven transporter. The length of the *yoeA* gene is 1392 bp, and it was predicted to encode a transmembrane protein with a molecular mass of 51.3 kDa. The NCBI BLAST results indicated that YoeA belongs to the multidrug and toxic compound efflux protein (MATE) family and shares 72.14% similarity with the YoeA protein from *Bacillus anthracis,* indicating that YoeA probably functions to secrete or efflux plipastatin into the environment.

To identify the function of YoeA, the *yoeA* gene was cloned and overexpressed in M-24 (Figure 3A,B), resulting in recombinant strain M-245 with a 2.84-fold enhancement of plipastatin production (1233 mg/mL) (Figure 3C). Compared with SrfP, YoeA showed a more effective increase in plipastatin production, providing preliminary evidence that YoeA could be the major plipastatin efflux protein. The growth curves of M-245 and M-246 were close to that of M-24, whereas the maximum OD_600_ of 4.63 was much higher than that of M-24 (OD_600_ = 3.84) (Figure 3D), indicating that overexpression of efflux transport proteins facilitates plipastatin and promotes cell growth, thereby increasing plipastatin production.

**Figure 5:**
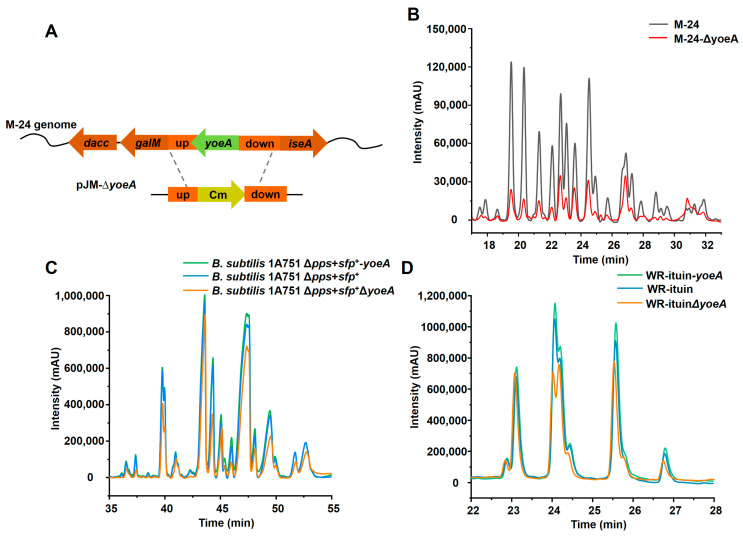
Effects of *yoeA* gene overexpression/knockout on the production of cyclic lipopeptides in *B. subtilis*. (**A**) Schematic representation of the construction of M-24Δ*yoeA*, a *yoeA* gene knockout strain. (**B**) HPLC profile of M-24Δ*yoeA*. (**C**) HPLC profiles of the effects of *yoeA* overexpression or knockout on surfactin production. (**D**) HPLC profiles of the effects of *yoeA* overexpression or knockout on iturin production.

To further confirm the efflux activity of YoeA, an M-24Δ*yoeA* mutant strain was generated by *yoeA* gene knockout (Figure 5A). Plipastatin production in this strain was reduced to 0.11 mg/mL, a 73.4% decrease compared with that of M-24 (Figure 5B). Plipastatin production was rescued and returned to the original level when *yoeA* was complemented in M-24Δ*yoeA*, further indicating that YoeA plays a major role as a plipastatin efflux transporter.

To explore the function of YoeA on other cyclic lipopeptides in *B. subtilis*, *yoeA* was overexpressed and knocked out sequentially in mono-producing surfactin (*B. subtilis 1A751Δpps + sfp^+^*) and iturin (WR-iturin) engineered strains. As shown in Figure 5C, overexpressing or knocking out *yoeA* slightly affected the surfactin yield, suggesting that the YoeA protein is not a major efflux protein for surfactin. For the WR-iturin strain, the production of only the C-15 and C-16 iturins increased slightly after *yoeA* overexpression, whereas the production of other iturins was unchanged. Knockout of *yoeA* also resulted in reduced production of the C-15 and C-16 iturins, with no change to the production of other iturins (Figure 5D). This suggests that YoeA is selective for specific types of iturin. These results demonstrate that YoeA is a specific transmembrane efflux protein for plipastatin.

### 3.4. Integrated Strain Engineering Pipeline for Enhanced Plipastatin Production

**Figure 6:**
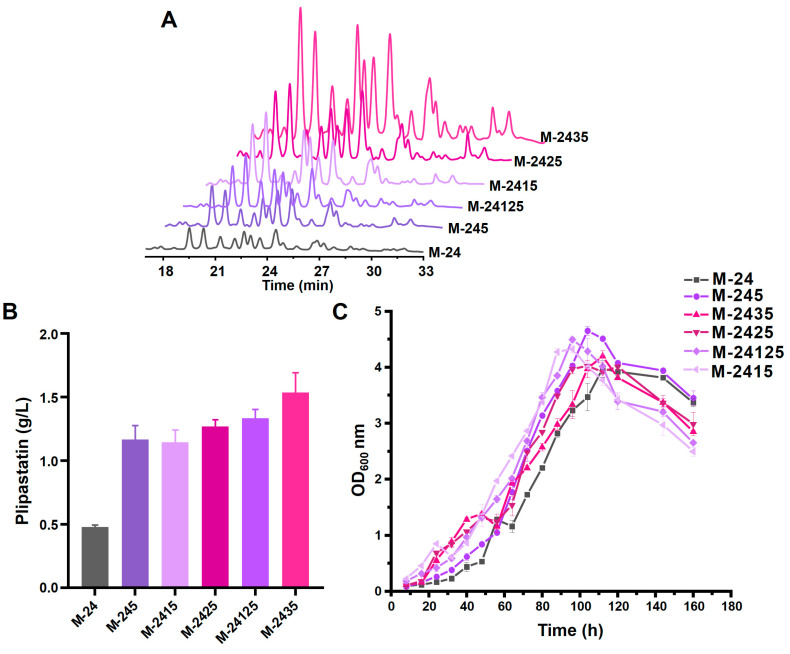
Effects of combinatorial gene overexpression on plipastatin production in recombinant strains. (**A**) The effect of recombinant gene overexpression strains in combination with *yoeA* overexpression on plipastatin production by HPLC. (**B**) Plipastatin production of recombinant overexpression strains in combination with *yoeA* overexpression. (**C**) Growth curve of recombinant overexpression strains in combination with *yoeA* overexpression.

To determine whether target genes have a synergistic effect on the improvement of plipastatin production, we iteratively overexpressed multiple genes associated with plipastatin synthesis by a combinatorial strain-engineering pipeline. The *lcfA* and *yoeA* genes were overexpressed in various combinations in the aforementioned promoter-replaced strains, resulting in seven recombinant strains (Table S1), and plipastatin production was analyzed by high-performance liquid chromatography (HPLC) (Figure 6A). M-2413, M-2423, and M-24123 were generated by overexpression of *lcfA* in M-241, M-242, and M-2412, respectively. These three recombinant strains produced higher levels of plipastatin than did M-24 (Figure S6B), but these levels were almost equal to the level of production when overexpressing *lcfA* alone, suggesting that an effective method for increasing plipastatin production is improving the loading level of fatty acids. Overexpression of *yoeA* in the same three strains generated M-2415, M-2425, and M-24125, of which the most productive strain, M-2425, yielded 1480 mg/mL of plipastatin, which was 1.53-fold higher than when overexpressing *yoeA* alone in M-24. This indicates that YoeA has a prominent effect in increasing plipastatin production. When the *lcfA* and *yoeA* genes were co-overexpressed in the M-24 strain to give strain M-2435, the plipastatin titer further improved to 1890 mg/mL, which was a 4.58-fold increase (Figure 6B).

The growth curves of these strains are shown in Figure 6C and Figure S6C. Except for M-24123 and M-2435, the time of maximum OD_600_ of the other strains was reduced by approximately eight hours compared with M-24. The maximum OD_600_ of all the strains increased upon co-overexpression of *yoeA*, indicating that YoeA enhances cellular density, which is significantly facilitated by the efflux of plipastatin. Based on these results, a higher plipastatin mono-production strain was constructed by simultaneous overexpression of *lcfA* and *yoeA*, and the resulting strain, M-2435, was selected to further explore iterative methods to increase plipastatin production.

### 3.5. Impact of AbrB Knockout on Plipastatin Production

**Figure 7:**
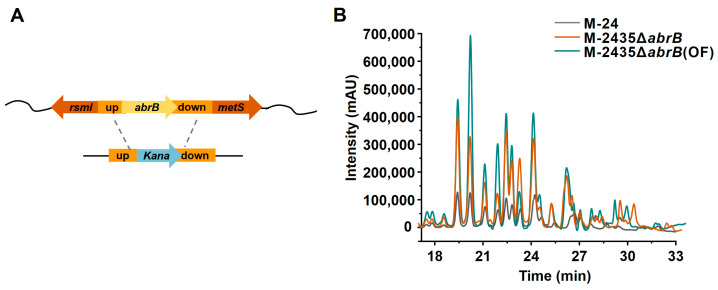
Effects of AbrB transcription factor knockdown and fermentation medium optimization on plipastatin production. (**A**) Schematic representation of the construction of the *abrB* gene knockout strain. (**B**) The effect of *abrB* gene knockdown and fermentation medium optimization on plipastatin production by HPLC, M-24Δ*abrB*35(OF) represents the plipastatin production of strain M-2435Δ*abrB*35 in optimized fermentation medium.

Antibiotic-resistant protein B (AbrB) is a global transcriptional regulator in *B. subtilis* that negatively controls the expression of a wide array of genes during exponential growth, such as the transcription of *spoVG*, *tycA*, and *aprE* [33]. AbrB can bind to target promoters to regulate gene expression in a concentration-dependent manner in *B. subtilis* [34]. For example, the *myc* operon is primarily regulated by AbrB, and knockout of *abrB* resulted in a fivefold increase in *myc* induction in *B. subtilis* ATCC 6633 [35]. A genome-wide binding profile of *B. subtilis* revealed that AbrB directly represses *ppsABCDE* in exponentially growing cells by an as-yet-unknown mechanism [36]. The *abrb* knockout strain was constructed as shown in Figure 7A. Knockout of *abrB* resulted in strain M-2435Δ*abrB*, which further increased plipastatin production to 2060 mg/mL, which is 4.31 times higher than the starting strain M-24 (Figure 7B). The AbrB transcription factor may bind to the P*_ppsA_* promoter sequence, leading to the inhibition of P*_ppsA_* transcription, and thus an *abrB* knockout would relieve the inhibitory effect on P*_ppsA_*, increasing P*_ppsA_* transcriptional activity and consequently enhancing plipastatin production.

The growth curve of the M-2435Δ*abrB* strain was consistent with M-2435 (Figure S8C), with the bacterial population entering the logarithmic phase at 56 h of incubation and reaching its maximum OD_600_ at 104 h. Although the OD_600_ value of M-2435Δ*abrB* was lower than M-2435, it was similar to the original strain M-24, and therefore the growth of the strain was not affected by *abrB* gene knockout.

### 3.6. Impact of Fermentation Optimization on Plipastatin Production

Modified Landy medium was selected as the original culture medium for M-2435Δ*abrB* to optimize fermentation parameters for plipastatin production. After optimization, the optimal culture medium composition was determined to be: 10 g L-Glu, 30 g glucose, 2.3 g sulfuric acid, 1 g potassium hydrogen phosphate, 0.5 g potassium chloride, 1.5 g magnesium sulfate, 1.6 mg sulfuric acid, 1.2 mg iron sulfate, 0.4 mg sulfuric acid, and MOPS 100 mM; pH 7.0–7.2. Under these conditions, the production of plipastatin was increased to 2514 mg/mL (Figure 7B), which is 5.26 times that of the initial strain M-24.

### 3.7. Evaluation of Antibacterial Properties of Plipastatin

**Figure 8:**
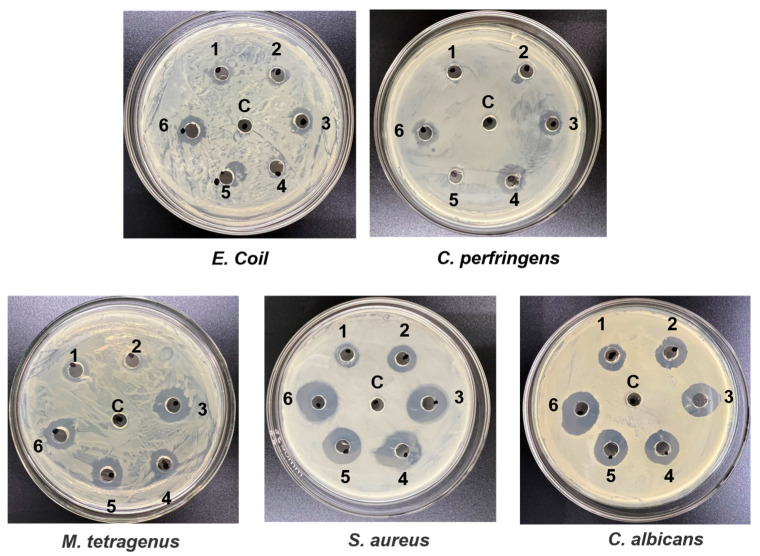
Antibacterial activities of crude extracts from the fermentation broth of different recombinant strains producing plipastatin. 1, 2, 3, 4, 5, and 6 represent the fermented extracts from M-24, M-2412, M-243, M-245, M-2435, and M-2435Δ*abrB*, respectively.

The antibacterial activity of plipastatin was assessed against a panel of pathogenic microorganisms, including *E. coli*, *S. aureus*, *C. albicans*, *M. tetragenus*, and *C. perfringens*. As shown in Figure 8, methanol was used as the control, and holes in plates of each pathogen were injected with fermented extracts from M-24(1), M-2412(2), M-243(3), M-245(4), M-2435(5), and M-2435Δ*abrB*(6). Of the tested pathogens, *S. aureus* was the most sensitive to plipastatin, followed by *C. albicans*, with inhibition zones of 28 mm and 25 mm, respectively. The maximum inhibitory diameters of the recombined strains were 8 mm against *E. coli* and 10 mm against *C. perfringens*. The inhibitory activity against *M. tetragenus* was plipastatin-concentration dependent, showing inhibitory zones ranging from 8 mm to 18 mm. These results suggest that fermentation extracts from M-24 and its derivative strains have strong antibacterial activity, consistent with previous reports, implying that plipastatin has great promise as an effective and eco-friendly antibacterial agent for applications in the post-harvest storage and preservation of fruits and vegetables.

## 4. Discussion

The plipastatin operon consists of five open reading frames (ORFs), including *ppsA*, *ppsB*, *ppsC*, *ppsD,* and *ppsE*, which are transcribed by the P*_ppsA_* promoter [35]. Substitution of the native plipastatin promoter in BMV11 with P*_veg_* led to a fivefold increase in plipastatin yield [14]. Plipastatin production was almost undetectable in wild Bs2500; however, after replacing the native plipastatin promoter with P*_amy_*, production was significantly increased to 434 mg/L [19]. In some cases, the same promoter can exhibit discrepant activities in different species. For example, the P*_fen_* promoter shows significantly higher activity in BBG21 than in BBG111 and FZB42. Replacement of the plipastatin promoter in BBG111 with P*_fen_* from BBG21 resulted in a ten-fold increase in plipastatin titers, underscoring the pivotal role of promoter replacement in enhancing plipastatin production [10]. In the present study, the activity of P_43_ was markedly stronger than those of P*_veg_* and P*_amy_* on comparison of β-galactosidase activity, and plipastatin production was increased to 607 mg/mL and 717 mg/mL when the natural promoters of the plipastatin operon and the *sfp* gene, respectively, were replaced.

Fatty acids are essential precursors for plipastatin production. Improvement of the fatty acid synthesis pathway will result in higher intracellular concentrations of metabolites associated with fatty acid anabolism, ultimately resulting in increased plipastatin production. The conversion of acetyl-CoA to malonyl-CoA is the rate-limiting step in fatty acid biosynthesis [37]. Overexpression of the *acs*, *birA*, and *accACD* genes promotes the synthesis of intracellular acetyl-CoA and malonyl-CoA and generates more precursors for fatty acid synthesis [38], consequently increasing plipastatin production from 10.43 mg/L to 24.7 mg/L [12]. YngH is an unconventional ACCase subunit, collaborating with carboxyltransferase to convert acetyl-CoA into malonyl-CoA in *B. subtilis* [39]. It displays high transcription levels in strains that produce significant amounts of surfactin [40]. However, in this study, overexpression of the *yngH* gene caused a 23% decrease in plipastatin production, and cell growth was also severely inhibited in M-24, indicating that *yngH* overexpression may disrupt the metabolic balance and impair normal growth, with a significant detrimental effect on plipastatin production.

The loading mechanism of fatty acid side chains in the non-ribosomal assembly of plipastatin remains poorly understood. The process may be analogous to that of surfactin because of their similar lipopeptide structure [41]. In the initial reaction of surfactin synthesis, 3-hydroxy fatty acids are activated by LcfA to form 3-hydroxy fatty acid-CoA thioesters, which are then recognized by the first condensation domain of surfactin synthase, SrfA, to initiate the loading of the fatty acid side chain. Therefore, LcfA might have a positive role in fatty acid loading [21,29]. Consistent with this, overexpression of the *lcfA* gene increased plipastatin production from 478 mg/mL to 980 mg/L.

Secondary metabolite production can be restricted by the accumulation of toxic products within the cell [34]. Efflux pumps are a general mechanism used by bacteria to transport intracellular metabolites out of the cell, thereby diminishing the accumulation of diverse molecules and preserving the normal physiological function of the cell [42]. Efflux pumps are primarily categorized into five families, based on their structure and function: the MFS (major facilitator superfamily), ABC (ATP-binding cassette superfamily), MATE (multidrug and toxic compound extrusion superfamily), RND (resistance-nodulation-cell division superfamily), and SMR (small multidrug resistance superfamily) families. Among these efflux pumps, only the ABC family requires ATP hydrolysis for energy provision [43]. *B. subtilis* produces three main families of cyclic lipopeptides, including plipastatins, surfactins, and iturins [44]. Currently, the efflux transport mechanisms of these lipopeptides are barely studied, with only preliminary research reported on the surfactin efflux transporter.

Li et al. reported that surfactin efflux in THY-7 was mainly dependent on PMF, describing experiments using liposomes and transmembrane transport inhibitors [23]. The three putative lipopeptide transporters—YcxA, KrsE, and YerP—that depend on PMF as an energy source were suggested to be involved in surfactin efflux. YerP belongs to the RND family and functions as the main surfactin efflux pump, and overexpression of *yerP* increased surfactin production by 145%. KrsE and YcxA are members of the MFS family, and overexpression of *krsE* increased surfactin production by 52%. However, the native *ycxA* gene in THY-7 has two base-pair deletions resulting in a frameshift mutation and loss of the surfactin transport function, and thus overexpression of the full-length *ycxA* gene without this mutation increased surfactin production by 89%.

The main efflux transporter for plipastatin is unknown, although it has been reported that the effect of plipastatin on lipids in biological membranes is concentration-dependent [45,46]. At low concentrations (<10 μM), plipastatin is dispersed as a monomer into the hydrophobic core of the model biomembrane, and this insertion of plipastatin does not significantly affect the phospholipid interfacial assembly. At sufficiently high plipastatin concentrations, the lipid bilayer is completely disrupted into mixed micelles [47]. This phenomenon is similar to the effects of surfactin on biological membranes, suggesting there may be a specific transporter for plipastatin efflux [48].

In general, lipopeptide operons include at least one putative transmembrane protein within or adjacent to the biosynthetic gene cluster (BGC). For example, the putative transporter *krsE* is situated within the kurstakin BGC [49], the bacitracin transporter *bcrABC* is located approximately 3 kb downstream of the bacitracin BGC [50], and the surfactin transporter *ycxA* is positioned 100 bp downstream of the surfactin BGC [23]. Consequently, we speculated that there may be plipastatin transporters in proximity to the plipastatin BGC.

An analysis of the flanking regions of the plipastatin BGC found that a presumed Na^+^-driven transporter gene, *yoeA*, was located upstream of the BGC. This protein exhibits 70.84% homology with the YoeA protein of the MATE family in *B. anthracis*. Overexpression of *yoeA* in M-24 increased the production of plipastatin 2.84-fold. Gene knockout and complementation experiments confirmed that YoeA is the major efflux transporter of plipastatin. To investigate the potential function of YoeA in the efflux of other lipopeptides, *yoeA* was either overexpressed or knocked out in mono-producing surfactin and iturin strains, which showed that YoeA has slight effects on the efflux. This is the first report to identify an efflux transporter specific to plipastatin, and further studies are needed to clarify the function of efflux proteins in plipastatin transmembrane transport.

The production of plipastatin is regulated by multiple transcription factors. AbrB, a global regulator, has been identified as a lipopeptide transcription inhibitor in *Bacillus* spp [33]. For example, AbrB can directly bind to the *bacABC* promoter and inhibit transcription of the bacitracin BGC in *B. licheniformis*, and *abrB* knockout resulted in a 17.5% increase in bacitracin production [51]. The plipastatin operon has been reported to be repressed by AbrB during the exponential growth phase, and a high transcription level was observed after the plipastatin promoter was fused with green fluorescent protein (GFP) [13]. In the present study, plipastatin production further increased to 2514 mg/mL, 5.26-fold higher than the starting strain, when *abrB* was knocked out, further supporting the conclusion that AbrB has a role in negative regulation as a global regulator of lipopeptides.

## 5. Conclusions

In summary, the native promoters of the plipastatin operon and the *sfp* gene were replaced by P43, increasing plipastatin production from 478 mg/mL to 607 mg/mL and 717 mg/mL, respectively. Overexpressing the *lcfA* gene enhanced plipastatin production to 980 mg/mL. A new Na^+^-driven efflux transporter, YoeA, which was identified as a MATE family member by transmembrane transport inhibitors, was shown to act as a specific efflux transporter for plipastatin through knockout and complementation experiments. Co-overexpression of *lcfA* and *yoeA* improved plipastatin production to 1890 mg/mL, which was further increased to 2060 mg/mL by knocking out the global negative regulatory factor AbrB. Finally, 2514 mg/mL plipastatin production was achieved after adding glutamic acid to the optimized culture medium, which was 5.26-fold higher than that of the initial strain. These results provide an effective integrated strategy to improve plipastatin production for industrial application, and the novel identification of the transport efflux protein YoeA contributes to our understanding of the mechanisms of plipastatin efflux.

## Figures and Tables

**Table 1:** Results of BLASTp analysis of the plipastatin biosynthesis gene cluster.

Protein	Size (aa)	Function Prediction	Accession Number
YngL	130	Integral inner membrane protein (*Bacillus velezensis*)	AEW31023.1
PpsABCDE	12408	Plipastatin synthetase	AEW31019.1 AEW31020.1 AEW31021.1 AEW31022.1 AEW31015.1
DacC	501	Penicillin-binding protein (*Bacillus velezensis*)	AEW31018.1
GalM	325	Putative epimerase (*Bacillus velezensis*)	AEW31017.1
YoeA	463	putative Na^+^-driven efflux transporter (*Bacillus velezensis)*	AEW31016.1
IseA	180	DL-endopeptidase inhibitor (*B. amyloliquefaciens* DSM 7)	WP_004399558.1

## Data Availability

The original contributions presented in the study are included in the article/Supplementary Material, further inquiries can be directed to the corresponding author.

## Supplementary Materials

The following supporting information can be downloaded at: https://www.mdpi.com/article/10.3390/foods13111785/s1 [52,53,54], Figures S1–S8: An integrated pipeline and overexpression of a novel efflux transporter, YoeA, significantly increase plipastatin production in *Bacillus subtilis*. Table S1, The strains used in this study; Table S2, The plasmids used in this study; Table S3, The primer sequences used in this study; Table S4, Experimental results of Box-Behnken designs for product yield; Table S5, Factor levels for response surface methodology; Table S6, Effects of carbon source on the diameter of the inhibition zone of M-2435Δ*abrb*; Table S7, Effects of glucose concentration on the diameter of the inhibition zone of M-2435Δ*abrb*; Table S8, Effects of amino acids on the diameter of the inhibition zone of M-2435Δ*abrb*; Table S9, Effects of glu concentration on the diameter of the inhibition zone of M-2435Δ*abrb*; Table S10, Effects of Inorganic Nitrogen Source on the Diameter of the Inhibition Zone of M-2435Δ*abrb*. Table S11, Effects of MgSO4 and KCl concentration on the diameter of the inhibition zone of M-2435Δ*abrb*; and Table S12, ANOVA of Quadratic Response Surface Model for Optimization Fermentation Medium.
