# Supplementary material for: An Integrated Pipeline and Overexpression of a Novel Efflux Transporter, YoeA, Significantly Increases Plipastatin Production in Bacillus subtilis
Source: Foods. 2024 Jun 6;13(11):1785. doi: 10.3390/foods13111785 (PMC11171584; doi:10.3390/foods13111785)
Supplement: Supplementary file 1 [file foods-13-01785-s001.zip › foods-2984552-supplementary-1.pdf]

Supplementary data

An Integrated Pipeline and Overexpression of a Novel Efflux Transporter, YoeA, Significantly Increases Plipastatin Production in *Bacillus subtilis*

Mengxi Wang, Jie Zheng, Sen Sun, Zichao Wu, Yuting Shao, Jiahui Xiang, Chenyue Yin, Rita Cindy Aye Ayire Sedjoah and Zhihong Xin *

Key Laboratory of Food Processing and Quality Control, College of Food Science and Technology, Nanjing Agricultural University, Nanjing 210095, China

* Correspondence: xzhfood@njau.edu.cn; Tel./Fax: +86-25-8439-5618

SUPPLEMENTARY TABLES AND FIGURES

Table S1. The Strains Used in This Study **.**
Table S2. The Plasmids Used in This Study.
Table S3. The Primer Sequences Used in This Study.
Table S4. Experimental Results of Box-Behnken Designs for Product Yield.
Table S5. Factor levels for Response Surface Methodology.
Table S6 Effects of Carbon Source on the Diameter of the Inhibition Zone of M-2435Δ*abrb*.
Table S7 Effects of Glucose Concentration on the Diameter of the Inhibition Zone of M-2435Δ*abrb*.
Table S8 Effects of Amino Acid on the Diameter of the Inhibition Zone of M-2435Δ*abrb*.
Table S9 Effects of Glu Concentration on the Diameter of the Inhibition Zone of M-2435Δ*abrb*.
Table S10 Effects of Inorganic Nitrogen Source on the Diameter of the Inhibition Zone of M-2435Δ*abrb*.
Table S11 Effects of $MgSO_4$ and KCl Concentration on the Diameter of the Inhibition Zone of M-2435Δ*abrb*.
Table S12 ANOVA of Quadratic Response Surface Model for Optimization Fermentation Medium.
Figure S1 PCR Validation of the Promoter Replacement Strain by PCR.
Figure S2 The Structure of Plipastatin.
Figure S3. PCR Validation of Recombination Strains with the Gene Overexpression.
Figure S4 PCR Validation of *yoeA* Gene-Knockout Strains.
Figure S5 Quantitative Analysis of Plipastatin by External Standard Method.
Figure S6 Effects of Gene Combination Overexpression on Plipastatin Production in Recombination Strains.
Figure S7 Effects of Knockout of the Transcription Factor AbrB on Plipastatin Production.
Figure S8 Bioinformatics Analysis of YoeA.

Table S1. The Strains Used in This Study.

Strain	Description	Source
E. coli DH5*α*	Competent cells	Tsingke Biotech Co.,Ltd
B. subtilis 1A751 M-24 (M-24)	*B. subtilis* 1A751 Δ*pps* + *pps* + *sfp* + *degQ* derivate: deleted part of surfactin biosynthetic gene cluster	[13]
M-241	M-24 derivate, P_{43}-*ppsA*	This study
M-242	M-24 derivate, P_{43}-*sfp*	This study
M-2412	M-24 derivate, P_{43}-*ppsA*, P_{43}-*sfp*	This study
M-243	M-24 derivate, *lacA::lcfA*	This study
M-244	M-24 derivate, *lacA::yngH*	This study
M-245	M-24 derivate, *lacA::yoeA*	This study
M-246	M-24 derivate, *lacA::srfP*	This study
M-2415	M-241 derivate, *lacA*::*yoeA*	This study
M-2425	M-242 derivate, *lacA::yoeA*	This study
M-24125	M-2412 derivate, *lacA::yoeA*	This study
M-2413	M-241 derivate, *lacA::lcfA*	This study
M-2423	M-242 derivate, *lacA::lcfA*	This study
M-24123	M-2412 derivate, *lacA::lcfA*	This study
M-2435	M-24 derivate, *lacA::yoeA-lcfA*	This study
M-24Δ*yoeA*	M-24 derivate, *yoeA*::Cm^R	This study
M-2435Δ*abrb*	M-2435 derivate, *abrb*::$Knan^R$	This study
B. subtilis 1A751 WR-*itu*(WR-itu)	Engineered iturin-mono-producing strain, *B. subtilis* 1A751 WR derivate, *amyE::ituDABC-sfp-degQ*	[52]
B. subtilis 1A751 Δ*pps* + *sfp*$^+$	Engineered surfactin-mono-producing strain, *B. subtilis* 1A751 Δ*pps* derivative, repairing *sfp* gene	[52]
WR-*itu-yoeA*	*B. subtilis* 1A751 WR- *itu* derivate, *lacA::yoeA*	This study
WR-*itu*Δ*yoeA*	*B. subtilis* 1A751 WR- *itu* derivate, ΔyoeA	This study
B. subtilis 1A751 Δ*pps* + *sfp*$^+$-*yoeA*	*B. subtilis* 1A751 Δ*pps* + *sfp*$^+$, *lacA::yoeA*	This study
B. subtilis 1A751 Δ*pps* + *sfp*$^+$Δ*yoeA*	*B. subtilis 1A751* Δ*pps* + *sfp*$^+$ derivate, ΔyoeA	This study

Table S2. The Plasmids Used in This Study.

Plasmids	Description	Source
pJOE8999	The gene-editing plasmid containing *Cas*9, gRNA scaffold, mannose-inducible promoter P_{man}, pUC origin of replication, and the temperature-sensitive replicon rep pE194ts; $Kana^R$	[53]
pJOE8999-P_{43}-*ppsA*	The plasmid pJOE8999 containing sgRNA, promoter P_{43}-*ppsA*; $Kana^R$	This study
pJOE8999-P_{43}-*sfp*	The plasmid pJOE8999 containing sgRNA, promoter P_{43}-*sfp*; $Kana^R$	This study
pJM-P_{ctc}-*lac*Z	The plasmid pJMP1 containing *LacZ gene*; Amp^R, Cm^R	Preserved in our laboratory
pJM-P_{43}-*lac*Z	The plasmid pJM-*lac*Z derivate; P_{43}-*lac*Z	This study
pJM-P_{veg}-*lac*Z	The plasmid pJM-*lac*Z derivate; P_{veg}-*lac*Z	This study
pJM-P_{sigA}-*lac*Z	The plasmid pJM-*lac*Z derivate; P_{sigA}-*lac*Z	This study
pJM-P_{xyl}-*lac*Z	The plasmid pJM-*lac*Z derivate; P_{xyl}-*lac*Z	This study
pJM-P_{manP}-*lac*Z	The plasmid pJM-*lac*Z derivate; P_{manP}-*lac*Z	This study
pJM-P_{gsi}-*lac*Z	The plasmid pJM-*lac*Z derivate; P_{gsi}-*lac*Z	This study
pJM-P_{ohrB}-*lac*Z	The plasmid pJM-*lac*Z derivate; P_{ohrB}-*lac*Z	This study
pJM-P_{aprE}-*lac*Z	The plasmid pJM-*lac*Z derivate; P_{aprE}-*lac*Z	This study
pJM-P_{rpsF}-*lac*Z	The plasmid pJM-*lac*Z derivate; P_{rpsF}-*lac*Z	This study
pJM-P_{43}-*yngH*	The plasmid pJM-*lac*Z derivate, P_{43}-*yngH*	This study
pJM-*lcfA*	The plasmid pJM-*lac*Z derivate, *lcfA*	This study
pJM-*srfP*	The plasmid pJM-*lac*Z derivate, *srfP*	This study
pJM-*yoeA*	The plasmid pJM-*lac*Z derivate, *yoeA*	This study
pJM-*yoeA-lcfA*	The plasmid pJM-*lac*Z derivate, *yoeA-lcfA*	This study
pJM-Δ*yoeA*	The plasmid pJM-*lac*Z derivate, Δ*yoeA*	This study
pJM-Δ*abrB*	The plasmid pJM-*lac*Z derivate, Δ*abrb*	This study

Table S3. The Primer Sequences Used in This Study.

Primers	Sequences (5′ - 3′)	
sfp-sgRNA-F	**TACG**AGAATGGCTGAAGAAGACAA	
sfp-sgRNA-R	**AAAC**TTGTCTTCTTCAGCCATTCT	
P*ppsA*-sgRNA-F	**TACG**AAACTGGGCGGCCGCTCCGC	
P*ppsA*-sgRNA-R	**AAAC**GCGGAGCGGCCGCCCAGTTT	
sfp-u-*F*	aa**GGCCAACGAGGCC**CGTTCCGCCACGAAATTG	*Sfi*I
sfp-u-*R*	aa**GGCCATGTTGGCC**GTTACAGATTCCGCGCCAA	
P_{43}-*sfp*-F	aa**GGCCAACATGGCC**AATCGATTCTTCAAAAGCTTCG	
P_{43}-*sfp*-R	CCATATATACTCCGTAAATCTTCATAAGCTTCTGTTATTAATTC	*Sfi*I
sfp-d-*F*	GAATTAATAACAGAAGCTTATGAAGATTTACGGAGTATATATGG	*Sfi*I
sfp-d-*R*	aa**GGCCTTATTGGCC**GAGTCGGTTGTTTCTCTG	*Sfi*I
Ts-F	GAGGTCATACTGGACATC	
Ts-R	CCATCAATCCATCACTGGT	
Vs-F	TACCGCAAACGGCAAAGTG	
Vs-R	AGCGTCCTTGGCCGCTTTA	
ppsA-u-*F*	aa**GGCCAACGAGGCC**CTGTCAATGGTTCAGATAC	*Sfi*I
ppsA -u-*R*	GCTTAATTGTTATCCGCTCA	
P_{43}-*ppsA*-F	aa**GGCCAACATGGCC**TCGATTCTTCAAAAGCTTCG	
P_{43}-*ppsA*-R	GAATTAATAACAGAAGCTTTTGAAGAACACTGTTTATTC	
ppsA-d-*F*	GAATAAACAGTGTTCTTCAAAAGCTTCTGTTATTAATTC	
ppsA-d-*R*	aa**GGCCTTATTGGCC**GGACAGCTCATTTGCCGT	*Sfi*I
Tp-F	GCAAGAACGTTGCTCGAG	
Tp-R	GTCAGACGGCTGATGCATA	
Vp-F	TGCGCTGTTCTCATTTGC	
Vp-R	CGTCAGCAAATGAGTTTGA	
yoeA F	gg**GGTACC**GACAGAATAAGTGCAATGGTTG	*kpnI*

yoeA R	cg**GAATTC**CTATTGGATGAGACGTGTG	*EcoRI*
lcfA F	gg**GGTACC**TCATTTGTCATTTCTGTCGGGC	*kpnI*
lcfA R	gg**GAATTC**TTAAGTTAACTTGAGCTCTTCAATCG	*EcoRI*
srfP F	GGGAGGCGCACACATACTAGATGAACCACGTTATTA ATTTCGTTCTGAAAAACA	
srfP R	ACCGGCGCTCAGCTGGAATTTTACTCTTCTTCCGTTCC CGGTT	
P_{43}-F	gg**GGTACC**TGATAGGTGGTATGTTTTCGC	*kpnI*
P_{43}-*yngH*-R	CTTATTTTTTTGCCAAAGCTGTAAATGTTTACAAAAGT ACTGATC	
P_{43}-*yngH*-F	GATCAGTACTTTTGTAAACATTTACAGCTTTGGCAAA AAAATAAG	
yngH R	gg**GAATTC**TTATAGGTGCTGTTTCAAAAAGTC	*EcoRI*
lcfA-*yoeA*-R	CAACCATTGCACTTATTCTGTCATAAGAAGGATTTTC AGGTTTGTC	
yoeA-*lcfA*-F	GACAAACCTGAAAATCCTTCTTATGACAGAATAAGT GCAATGGTTG	
V-*yoeA*-F	AATGCAACAGCTGCATACG	
V-*lafA*-F	TACATCAGGTACGACCGG	
V- *srfP*-F	TCGATAAGCTTGACCACCC	
V-R	CATATACATTGCCCGTCGGT	
pJM F	CCCATCATTCTTGAAGACGAAAG	
pJM R	CACCCCATCCTGATATTGTC	
dely-d-F	GACAATATCAGGATGGGGTGGCGGATAATGCCAACT GTAATGCT	
dely-d-R	ACCAATCGAAGCGGTACCAATCGAACTTCCTGAACC GATC	
Cm-F	TTGGTACCGCTTCGATTGGTACCGCTGCTTAAAG	
Cm-R	CCAGCTTGAAAATACCCAGCTTGAAATCGATATCTCT GC	
dely-u-F	GCTGGGTATTTTCAAGCTGGGTATTATCAATTCGTCT	
dely-u-R	TCGTCTTCAAGAATGATGGGGGCATGATGAGGAAAT TGCTTCTG	
V-d-F	GCGAGAAGAATCATAATGGGG	
V-d-R	GCCTTGTGTATCAAGGGTTAC	

V-u-F	CGCGACTGCAGAGATATCG
V-u-R	CCAACTGATCTTCAGCATC
V-dely-F	GTGCCTAGATCACTGCACATG
V-dely-R	GGAGTCCAAATACCAGAGAATG
dela-d-F	CGGCTGGATATTAGTTCTTATAGTCAGTAATGTCTTGGA
dela-d-R	CCATGATTACGCCAAGCTGCATCTCCTGCGACTG
Ka-F	AAGCAATAACTGACCCCGGATGAATG
Ka-R	AGAACTAATATCCAGCCGGCGTCCCGG
dela-u-F	TACTGAGAGTGCACCATAACGATTTCTGATCCCGG
dela-u-R	CGGGGTCAGTTATTGCTTAATATGGTAAGCATCGTAC
YZU R	CCAACTGATCTTCAGCATC
YZ ORI R	GGTTTCGCCACCTCTGAC
V-dela-F	CAGTGGATGCGATTGCTGCG
V-dela-R	GGAGTCCAAATACCAGAGAATG

Note: The lowercase bases represent protected bases, the bold bases represent the restriction enzyme site, and the underlined bases represent the homologous sequences for overlapping PCR or infusion clones.

Figure S1 PCR validation of the promoter replacement strain by PCR. (A) The gel electrophoresis image of recombinant strain M-241, the length of the left and right junction are 1196 bp and 1328 bp. (B) The gel electrophoresis image of recombinant strain M-242, the length of the left junction is 2664 bp.

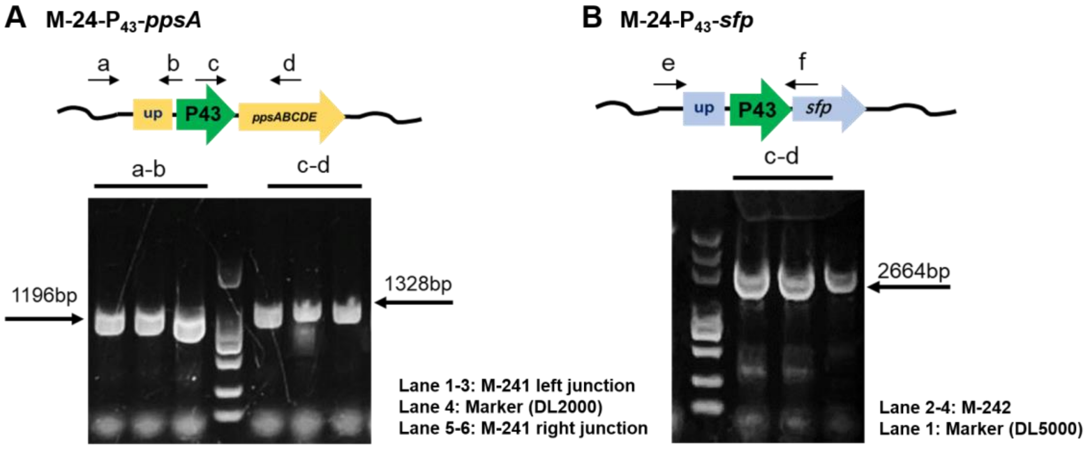

Figure S2 The Structure of Plipastatin.

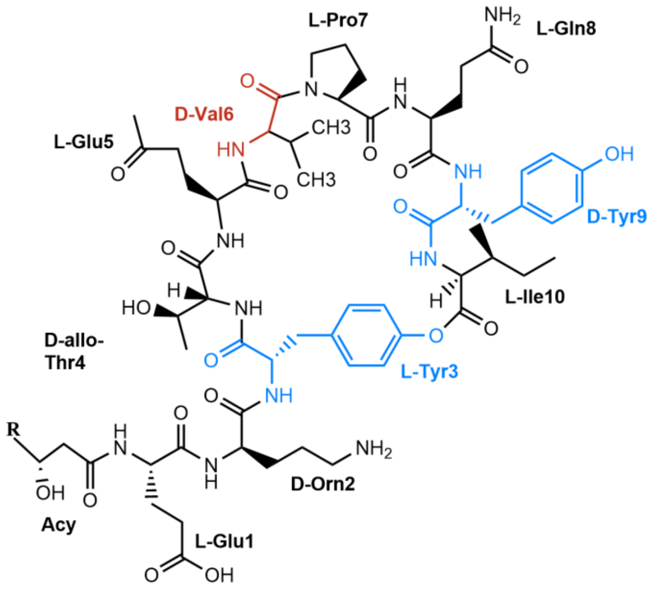

Figure S3 PCR Validation of Recombination Strains with the Gene Overexpression. (A) The gel electrophoresis image of the overexpression *lcfA* strains, the length of PCR production is 2212 bp. (B) The gel electrophoresis image of the overexpression *yngH* strains, the length of PCR production is 1913 bp. (C) The gel electrophoresis image of the overexpression *srfP* strains, the length of PCR production is 1825 bp. (D) The gel electrophoresis image of the overexpression *yoeA* strains, the length of PCR production is 1638 bp.

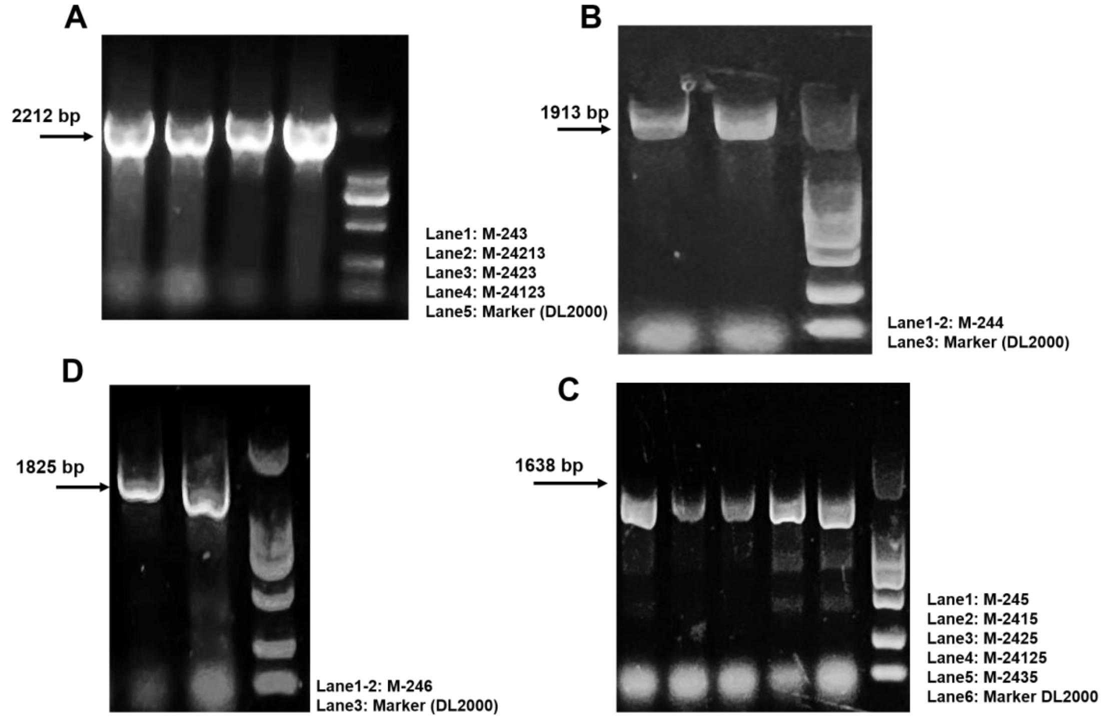

Figure S4 PCR Validation of *yoeA* Gene-Knockout Strains. (A) The gel electrophoresis image of the amplification of *yoeA* gene and positive clones show no bands. (B) The gel electrophoresis image of *yoeA*-knockout strains, the length of PCR production is 1475 bp.

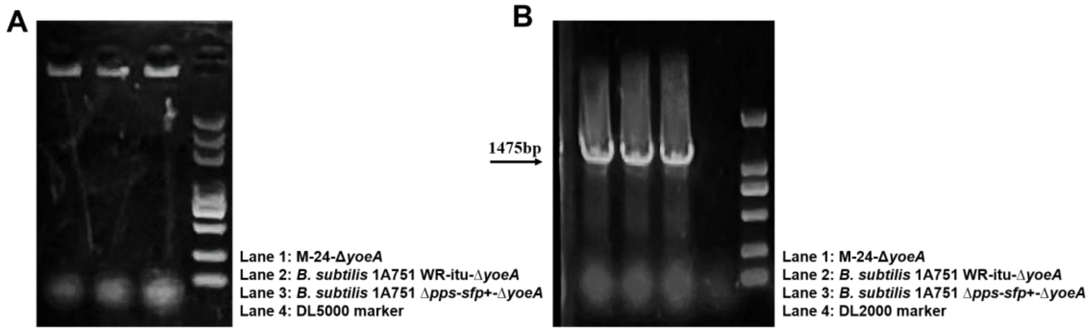

Figure S5 Quantitative Analysis of Plipastatin by External Standard Method. (A) Isolation, purification, and preparation of the plipastatin of M-24. (B) HPLC profile at different concentrations of the purified plipastatin1. (C) The MALDI-TOF diagram of C17-plipastatin B(plipastatin 1)[54].

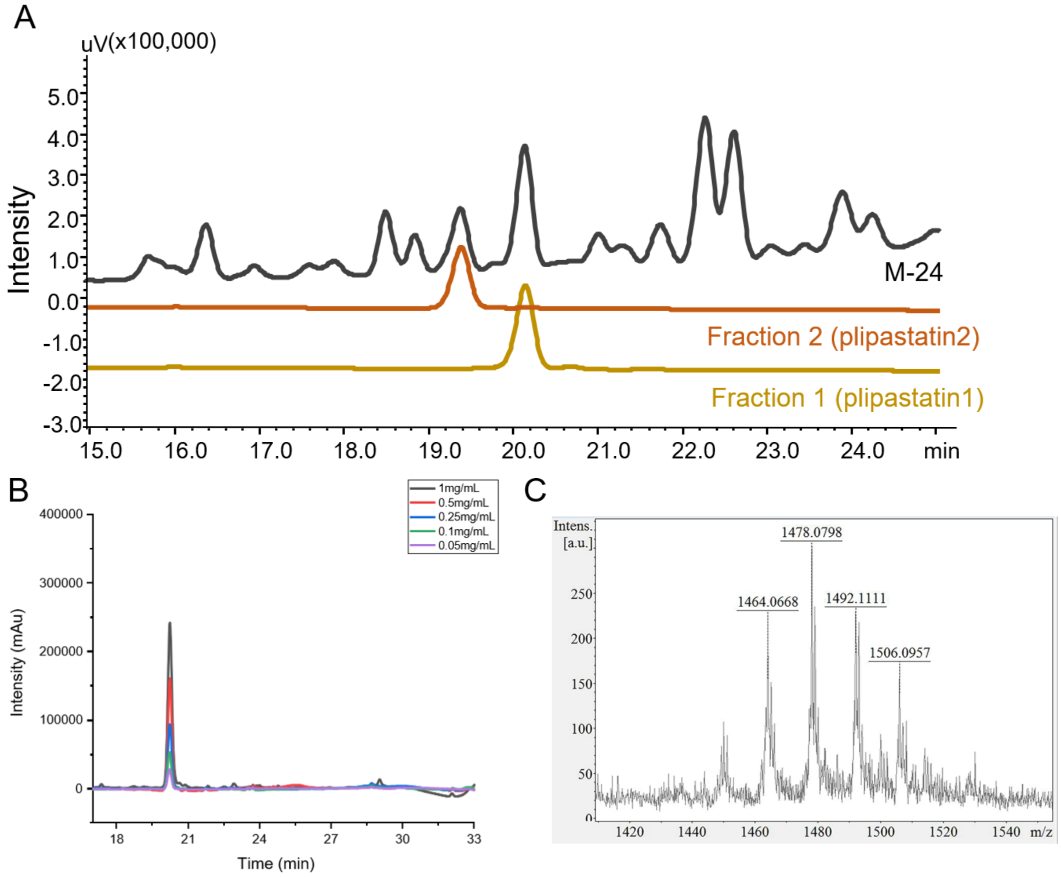

Fig. S6. Effects of Gene Combination Overexpression on Plipastatin Production in Recombination Strains. (A) The effect of overexpression combined with LcfA on plipastatin production by HPLC. (B) plipastatin production of recombinant strains overexpressing in combination with *lcfA* gene. (C) Growth curve of overexpression strain combined with *lcfA* gene.

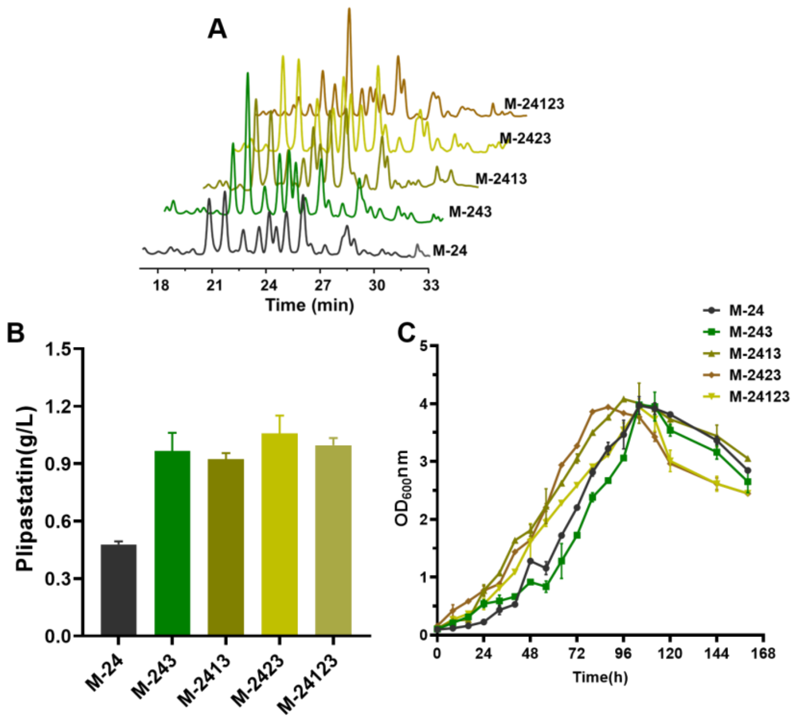

Figure S7 Effects of Knockout of the Transcription Factor AbrB on Plipastatin Production. (A) The effect of *abrb* gene knockout on plipastatin production by HPLC. (B) plipastatin production of the *abrb* gene knockout strain. (C) Growth curve of the *abrb* gene knockout strain.

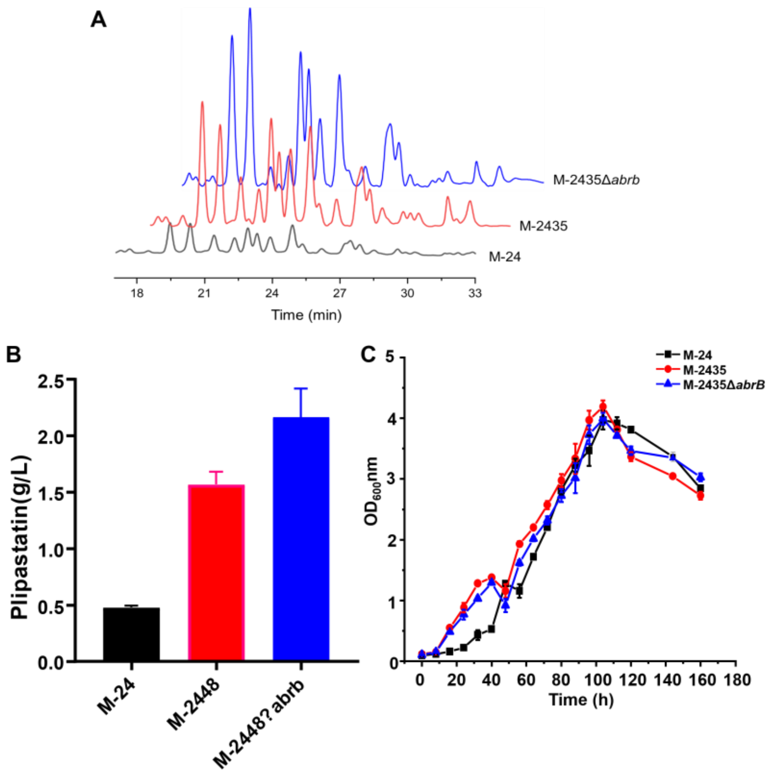

Figure S8 Bioinformatics Analysis of YoeA. (A) YoeA transmembrane segment was predicted using TMHMM. (http://www.cbs.dtu.dk/services/TMHMM-2.0/) (B) 3D structure prediction of YoeA by AlphaFold.

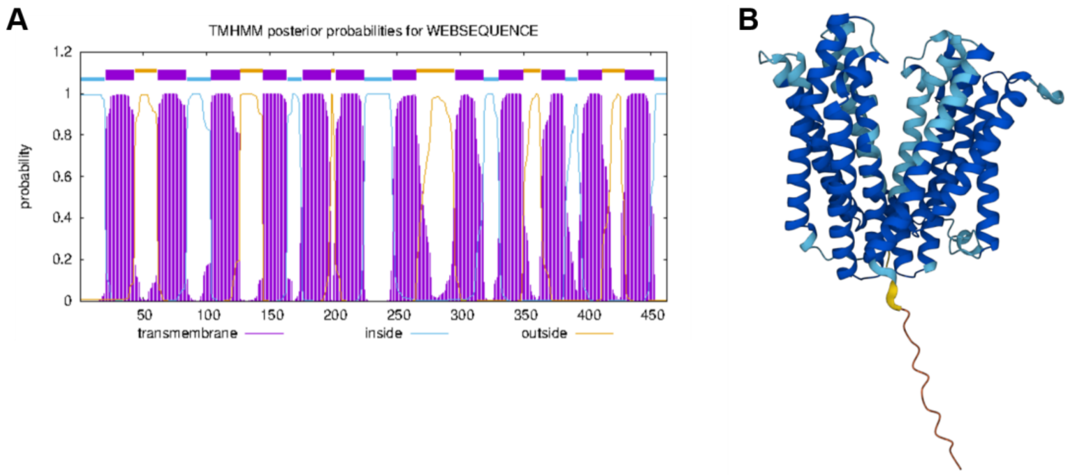

1. Optimization of fermentation medium

1.1. Methods

It has been reported that the modified Landy medium is more favorable to the production of plipastatin[16], the carbon source, nitrogen source, and amino acid were further optimized in this medium. The carbon source in the fermentation medium was optimized by the diameter of the inhibition zone as an indicator. Glucose, maltose, sucrose, α-lactose, mannose, and fructose were used as the carbon source for the fermentation medium, and after selecting the best carbon source, the carbon source content (10.00, 20.00, 30.00, 40.00, 50.00, 60.00, 70.00 g/L) to determine the optimal carbon source concentration.

The nitrogen source in the fermentation medium was optimized by the same method. $(NH_4)_2SO_4$ in the primary culture medium was replaced by urea, NH_4HCO_3, and NH_4Cl with equivalent proportions to determine the optimal nitrogen source.

The amino acid was optimized in the fermentation medium by the same method. L-Glutamic (L-Glu), L-serine (L-Ser), L-asparagine (L-Asn), L-tyrosine (L-Tyr), L-ornithine (L-Orn), L-threonine (L-Thr), L-proline (L-Pro), L-glutamine (L-Gln), L-isoleucine acid (lle) were added into fermentation medium, after obtaining the optimal amino acid and the addition amount (1.00, 3.00, 5.00, 7.00, 9.00, 11.00, 13.00 g/L) was further optimized.

The inorganic salts $MgSO_4$ and KCl were optimized by the same method with different concentrations respectively (0.00、0.50、1.00、1.50、2.00、2.50、3.00 g/L).

Based on the results of the single-factor experiment, a response surface experiment coupled with the Box-Behnken design was employed to optimize the medium (**Table S4**), with the diameter of the inhibition zone as the response value. A three-factor, three-level experiment (**Table S5**) was designed using L-Glu(A), $MgSO_4$ (B), and KCl (C) as variables, which were found to have a significant impact on plipastatin production. Design Expert 8.0.6 software performed quadratic regression fitting on experimental data to derive the optimal formula for the culture medium. L-glutamic acid 10 g/L, glucose 30 g/L, potassium dihydrogen phosphate 1 g/L, potassium chloride 0.5 g/L, magnesium sulfate heptahydrate 1.5 g/L, copper sulfate 1.6 × 10^{-3} g/L, ferric sulfate 1.2 × 10^{-3} g/L, manganese sulfate 0.4 × 10^{-3} g/L, 100 mM MOPS, pH 7.0-7.2.

Table S4. Experimental Results of Box-Behnken Designs for Product Yield.

Number	Glu (g/L)	$MgSO_4$ (g/L)	KCl (g/L)	Diameter of the inhibition zone （cm）
1	9.00	1.50	1.00	0.89
2	9.00	1.50	1.00	0.91
3	9.00	1.00	1.50	0.69
4	9.00	1.50	1.00	0.92
5	9.00	2.00	1.50	0.8
6	11.00	2.00	1.00	0.92
7	11.00	1.00	1.00	0.84

8	7.00	1.00	1.00	0.75
9	9.00	2.00	0.50	0.87
10	7.00	1.50	0.50	0.9
11	9.00	1.50	1.00	0.94
12	11.00	1.50	0.50	0.97
13	9.00	1.50	1.00	0.95
14	9.00	1.50	0.50	0.82
15	7.00	1.50	1.00	0.84
16	7.00	1.50	1.50	0.81
17	11.00	1.50	1.50	0.87

Table S5. Factor levels for Response Surface Methodology.

Factors	level		
	-1	0	1
A: Glu（g/L）	7	9	11
B: $MgSO_4 \cdot 7H_2O$（g/L)	1	1.5	2
C: KCl（g/L)	0.5	1	1.5

1.2. Results

Based on the modified Landy medium, the carbon source of the medium was optimized and the antibacterial activity of the fermentation extract was used as an indicator. The results showed that glucose was effective in increasing the production of plipastatin and enhancing the antibacterial activity. When the glucose concentration is 30 g/L, the diameter of the inhibition zone reaches 0.98 cm.

Table S6. Effects of Carbon Source on the Diameter of the Inhibition Zone of M-2435Δ*abrb*.

Carbon source	Glucose	maltose	sucrose	α-lactose	mannose	fructose
Diameter of the inhibition zone (cm)	**0.96±0.07**	0.5±0.05	0.8±0.04	0.7±0.02	0.68±0.02	0.71±0.02

Table S7. Effects of Glucose Concentration on the Diameter of the Inhibition Zone of M-2435Δ*abrb*.

Glu（g/L）	10	20	30	40	50	60	70
Diameter of the inhibition zone (cm)	0.72 ±0.03	0.85 ±0.04	**0.90±0.03**	0.85±0.03	0.82±0.03	0.80±0.03	0.79±0.03

Based on the modified Landy medium, the carbon source of the medium was optimized and the antibacterial activity of the fermentation extract was used as an indicator. The results showed that glucose was effective in increasing the production of plipastatin and enhancing the antibacterial activity. When the glucose concentration is 30 g/L, the diameter of the

inhibition zone reaches 0.98 cm.

Table S8. Effects of Amino acid on the Diameter of the Inhibition Zone of M-2435Δ*abrb*.

Amino acid	Glu	Tyr	Orn	Thr	Pro	Gln	lle
Diameter of the inhibition zone (cm)	**0.96±0.07**	0.5±0.05	0.8±0.04	0.7±0.02	0.68±0.02	0.71±0.02	0.70±0.01

Table S9. Effects of Glu Concentration on the Diameter of the Inhibition Zone of M-2435Δ*abrb*.

Glu（g/L）	1	3	5	7	9	11	13	15
Diameter of the inhibition zone (cm)	0.63±0.02	0.75±0.05	0.90±0.03	0.93±0.03	**0.96±0.03**	0.92±0.03	0.88±0.03	0.84±0.04

To determine the most efficient amino acid for the production of plipastatin, the seven precursor amino acids of plipastatin including L-Ser and L-Asn were added to the culture medium. The results showed that when L-Glu was added at 9 g/L (**Table S7 and S8**), the diameter of the inhibition zone was significantly increased.

Table S10. Effects of Inorganic Nitrogen Source on the Diameter of the Inhibition Zone of M-2435Δ*abrb*.

Inorganic nitrogen source	$(NH4)_2SO_4$	NH_4HCO_3	NH_4Cl	urea
Diameter of the inhibition zone (cm)	0.65±0.03	0.72±0.03	0.73±0.02	0.38±0.07

The nitrogen sources including urea, NH_4HCO_3, and NH_4Cl were determined for producing plipastatin and the results showed that NH_4Cl was more efficient for producing plipastatin than other nitrogen sources.

Table S11. Effects of $MgSO_4$ and KCl Concentration on the Diameter of the Inhibition Zone of M-2435Δ*abrb*.

Concentration (g/L)	$MgSO_4$•$7H_2O$	KCl
0	0.52±0.03	0.52±0.03
0.5	0.81±0.02	0.82±0.03
1	0.89±0.02	0.91±0.04
1.5	0.92±0.03	0.84±0.04
2	0.86±0.04	0.74±0.04
2.5	0.83±0.03	0.65±0.03

The inorganic salts $MgSO_4$ and KCl with different concentrations were also optimized for producing plipastatin, the results showed that $MgSO_4$•$7H_2O$ at 1.5 g/L, KCl at 1 g/L could improve the activity of the fermentation extract.

Based on the above results, L-Glu(A), $MgSO_4$(B), and KCl(C) were selected for further optimization according to the Box-Behnken central combination design principle. As shown in

Table S6, the P-value of the three factors was <0.01, indicating extremely significant effects of all three, where the degree of effect was L-Glu > $MgSO_4 \bullet 7H_2O$ > KCl. The regression equation was Y=0.92+0.038A+0.041B-0.049C-2.500E-003AB-2.500E-003AC+0.015BC+4.000E-003A2-0.089B2-0.038C2, and the optimal composition was L-Glu 10 g, glucose 30 g, sulfuric acid 2.3 g, potassium hydrogen phosphate 1 g, potassium chloride 0.5 g, magnesium sulfate1.5 g, Cupric sulfate 1.6 mg, ferric sulfate 1.2 mg, manganese Sulfate 0.4 mg, MOPS 100 mM, pH to 7.0-7.2.

Table S12. ANOVA of Quadratic Response Surface Model for Optimization Fermentation Medium.

Sources	Sum of Squares	df	Square	Value	Prob > F	Significant
Model	0.086	9	9.52E-03	26.61	0.0001	Significant
A-L-Glu	0.011	1	0.011	31.44	0.0008	
B-$MgSO_4 \cdot 7H_2O$	0.014	1	0.014	38.04	0.0005	
C-KCl	0.019	1	0.019	53.13	0.0002	
AB	2.50E-05	1	2.50E-05	0.07	0.7992	
AC	2.50E-05	1	2.50E-05	0.07	0.7992	
BC	9.00E-04	1	9.00E-04	2.51	0.1568	
A^2	6.74E-05	1	6.74E-05	0.19	0.6774	
B^2	0.033	1	0.033	92.15	< 0.0001	
C^2	6.24E-03	1	6.24E-03	17.44	0.0042	
Residual	2.51E-03	7	3.58E-04			
Lack of Fit	2.25E-04	3	7.50E-05	0.13	0.9363	Not Significant
Pure Error	2.28E-03	4	5.70E-04			
Cor Total	0.088	16				
